# Attention Deficit Predicts Intellectual Functioning in Children with Neurofibromatosis Type 1

**DOI:** 10.1155/2019/9493837

**Published:** 2019-12-10

**Authors:** Magdalena Heimgärtner, Sofia Granström, Karin Haas-Lude, Robert A. Leark, Victor-Felix Mautner, Karen Lidzba

**Affiliations:** ^1^University Children's Hospital Tübingen, Department of Pediatric Neurology, Hoppe-Seyler-Straße 1, 72076 Tübingen, Germany; ^2^University Hospital Hamburg-Eppendorf, Department of Neurology, Martinistraße 52, 20246 Hamburg, Germany; ^3^Californian School of Forensic Studies, Alliant International University San Diego, 10455 Pomerado Road, San Diego, CA 92131, USA; ^4^Children's University Hospital, Division of Neuropediatrics, Development and Rehabilitation, Bern University Hospital, Inselspital, Freiburgstrasse, 3010 Bern, Switzerland

## Abstract

**Aims:**

Attention deficit hyperactivity disorder (ADHD) is one of the most frequent neurocognitive impairments in neurofibromatosis type 1 (NF1) and a well-known risk factor for intellectual dysfunction in general. Since NF1 is per se associated with intellectual difficulties, this comorbidity may be crucial for the cognitive development of affected patients. In our study, we investigated if attention deficits are associated with intellectual functioning in NF1 and if children with NF1 plus ADHD differ in their intellectual and attention profiles from children affected by NF1-only or ADHD only.

**Methods:**

111 children aged between 6 and 12 years (53 NF1 plus ADHD, 28 NF1-only, 30 ADHD-only) performed the German version of the intelligence test WISC-IV and a continuous performance test (T.O.V.A.) to assess attention functions. Parents completed questionnaires about everyday attention and executive functions (Conners 3®, BRIEF).

**Results:**

Children with NF1 plus ADHD showed significantly lower intelligence test scores (full-scale IQ: 89.39 [1.40]) than patients with NF1-only (full-scale IQ: 101.14 [1.98]; *p* < .001), and intellectual functioning correlated significantly with attention performance in NF1 (*p* < .001). As compared to NF1-only, attention, and executive functioning were impaired on several dimensions (T.O.V.A., Conners 3® and BRIEF) in NF1 plus ADHD. ADHD-only was associated with significantly higher problem scores regarding hyperactivity/impulsivity and inattention (Conners 3®). NF1-only was associated with inattentiveness when compared to the normative sample of the T.O.V.A.

**Conclusion:**

NF1 is associated with variable attention problems. Severe attention deficits appear to be a risk factor for intellectual dysfunction in NF1, more than NF1 without attention deficit. NF1 plus ADHD presents a specific cognitive profile, which differs from that of NF1 and from neurotypical ADHD.

## 1. Introduction

Neurofibromatosis type 1 (NF1) is an autosomal dominant single-gene disorder, affecting the nervous system. It has an incidence rate of one in 2600–3000 individuals. While approximately 50% of NF1 cases are caused by an inherited defect of the NF1 gene, the remainder traces back to spontaneous mutations [1].

NF1 is characterized by a wide range of physical complications [1] and is considered to cause a variety of cognitive dysfunctions [2]. Apart from slightly decreased intelligence scores, academic underachievement (e.g., writing, reading, spelling, and arithmetic), language problems, memory deficits, impaired visuospatial abilities, and executive problems [3], serious attention problems are one of the most prominent cognitive features in NF1 [2]. Over and above unspecific attention problems, up to 50% of all NF1 patients present ADHD-like symptoms and fulfil the diagnostic criteria for attention deficit hyperactivity disorder (ADHD) [2, 4–6] according to the *Diagnostic and Statistical Manual of Mental Disorders* (DSM-IV) [7]. Since the ADHD-diagnosis is based exclusively upon behavioral criteria, ADHD-like symptoms in NF1 may consequentially be diagnosed as ADHD, even if etiology and developmental course of these symptoms are unknown so far and the high prevalence does not suggest a simple comorbidity.

The etiology of attention deficits in NF1-associated ADHD must be assumed to differ from that in ADHD in neurotypical children without NF1 (ADHD-only). While genetic factors seem to explain a high percentage of phenotype variance in ADHD-only [8], the incidence of attention problems in patients with NF1 is far higher than in their healthy siblings or parents [4], arguing against an independent heredity of ADHD in NF1. Although probably situated at the endpoint of different genetic pathways, the attention deficits in both groups seem to be generated by a disturbance of the catecholaminergic metabolism in fronto-striatal brain structures. Recent studies suggest that neurofibromin deficiencies lead to reduced dopamine signaling, which might be responsible not only for impairments in learning and memory [9], but even more so for attention problems in NF1 [10]. In a mouse model, Brown and colleagues could trace attention defects in NF1-mutant mice back to reduced dopamine levels and reduced postsynaptic dopamine signaling in the striatum. Striatal dopamine levels could be normalized by dopamine-elevating drugs (e.g., methylphenidate), accompanied by an amelioration of attention performance [11]. First publications on children with NF1 plus ADHD (NF1^ADHD^) provide evidence that, similar as in ADHD-only, pharmaceutical interventions with methylphenidate (MPH) have positive effects on the core symptoms of ADHD [17]. Specifically in patients with NF1^ADHD^, there is tentative evidence that MPH might even lead to intellectual improvement [11, 12].

Regarding their cognitive profile, it is well known that patients with NF1 do not represent a homogeneous group [5]. There seem to be some combinations of cognitive dysfunctions, which have more impact than others. Especially attention deficit and ADHD appear to increase the risk for a number of other cognitive comorbidities, like deficits in full-scale, verbal, and performance-related IQ [13], functional executive abilities, sustained attention, receptive language, and academic ability (reading, spelling, and mathematical reasoning) [14]. The general profile of comorbidities is not too different from that of patients with ADHD-only, who may, among other conditions, also suffer from various learning disabilities and specific language impairment [15].

Similar to patients with ADHD-only [15], patients with NF1^ADHD^ score significantly lower in intelligence tests than healthy (sibling-) controls [14–16] and show lower IQ scores than NF1 patients without ADHD (NF1-only) [4, 13, 14, 17]. Also, specific learning disabilities and academic underachievement are associated with ADHD in children, both with and without NF1 [14, 16]. The course and profile of cognitive impairments seems stable for NF1^ADHD^ as well as for ADHD-only [16, 18, 19], mediated, however, by the presence or absence of T2 signal hyperintensities on MRI for NF1 [20]. Executive dysfunctions seem to be a hallmark of both, ADHD-only and NF1^ADHD^, including deficits in inhibition, sustained attention, verbal fluency, and especially working memory deficits, which are well described in both populations [21, 22].

So far, few studies directly compared children with NF1^ADHD^, NF1-only, and ADHD-only [6, 17]. Potvin and colleagues retrospectively compared the intellectual profile of children with NF1 with and without ADHD and children with ADHD-only. They found that children with NF1 demonstrate an uneven intellectual profile with preserved verbal abilities and reduced nonverbal abilities, working memory, and processing speed. In addition, the combination of NF1 and ADHD led to lower performances in most areas of the intelligence test WISC-IV than ADHD or NF1 exclusively [17].

Furthermore, the specific profile of attention functions seems to differ between NF1^ADHD^ and ADHD-only: In a treatment study by Mautner and colleagues, children with NF1^ADHD^ seemed especially impaired in impulse control, whereas children with ADHD-only displayed more deficits in sustained attention. Both groups, however, were impaired in response time and variability of response time [6]. A more recent study by Lion-François and colleagues found fundamental qualitative and quantitative differences between the attention deficits of NF1^ADHD^ and ADHD-only. Children with NF1^ADHD^ showed lower overall performances in the areas of intensive, selective, and executive attention, while children with ADHD-only showed slower response times in a sustained attention task. The authors conclude that the condition NF1^ADHD^ is not only the sum of NF1 plus ADHD, and that ADHD symptomatology does not contribute to all attention deficits of patients with NF1 [23]. To back this assumption, however, a NF1 control group without ADHD would be needed. Altogether, there is very little literature on this particular topic and more studies are needed to elucidate differences and commonalities of the intellectual and attention profiles of children with NF1^ADHD^, NF1-only, and ADHD-only.

The aim of our study was to compare the intellectual and the attention profile of children with NF1^ADHD^, NF1-only, and ADHD-only in a prospective approach. Our study may help to substantiate NF1-typical cognitive characteristics and to elaborate differences between NF1^ADHD^ and NF1-only, as well as NF1^ADHD^ and ADHD-only:

(1) Firstly, we assumed that ADHD symptoms are a specific risk factor for intellectual functioning in NF1. Therefore,
  (a) we expected patients with NF1-only and ADHD-only to perform significantly better on measures of intellectual functions than patients with NF1^ADHD^. Additionally,
  (b) we expected intellectual performance to be correlated with attention performance in patients with NF1.
(2) Regarding the attention profile, we expected significant differences between NF1-only, NF1^ADHD^, and ADHD-only on measures of inattention, hyperactivity, impulsivity, and executive functions.

To this end, we recruited patients within the normal and borderline range of intelligence, so that the sample would be representative for NF1 and ADHD regarding intelligence.

## 2. Material and Methods

### 2.1. Participants

Three groups of patients were recruited for this study: (1) patients with NF1^ADHD^, (2) patients with NF1 without ADHD (NF1^control^), and (3) patients with neurotypical ADHD without NF1 (ADHD^control^). Participants for the NF1 groups were consecutively recruited between 2013 and 2016 at the University Children's Hospital Tübingen and the University Medical Center Hamburg-Eppendorf. For the ADHD^control^ group, all participants were recruited at the University Hospital Tübingen. All participants were native speakers of the German language. NF1 was diagnosed according to the National Institute of Health Consensus Development Conference statement [24], ADHD was diagnosed according to DSM-IV, based on a standardized parent interview by a psychologist, questionnaires (Disyps-KJ) [25] and clinical impression (observation of the child's behavior during the assessment). Importantly, diagnosis was made independently from any test results. Typical psychiatric comorbidities of ADHD and NF1 were allowed, e.g. specific developmental disorders of speech, language, scholastic skills, motor function, conduct disorders or emotional disorders (recorded comorbidities and their frequencies in our patient groups are listed in Table 1). Patients with evidence for neurological diseases with intracranial manifestations (symptomatic optic nerve glioma, brain tumor, traumatic brain injury, stroke), or with any form of epilepsy, very preterm birth or severe psychiatric disorders (e.g., autism spectrum disorders) were excluded.

Altogether, 126 children, aged between 6 and 12 years, were recruited. Fifteen participants were excluded after the assessment because of comorbid autistic disorders (1 NF1^ADHD^, 1 NF1^control^, 4 ADHD^control^), falling outside the predefined range of intelligence (IQ 70–115; 0 NF1^ADHD^, 1 NF1^control^, 4 ADHD^control^), or—in the ADHD^control^ group—for no longer meeting the diagnostic criteria for ADHD at the time of the assessment (4 participants). This left 53 children in the NF1^ADHD^ group, 28 in the NF1^control^ group, and 30 in the ADHD^control^ group for analysis. Thirteen participants with NF1 had asymptomatic optic nerve glioma in the absence of other intracranial pathology as evidenced by MRI. Nine participants (7 NF1^ADHD^, 2 ADHD^control^) received stimulant medication prior to the study, but not on the assessment day, allowing a wash out period of ≥24 hours. Approval of the local ethics review board (655/2012BO1), patients' assent and written informed consent of their caregivers were obtained prior to the investigations.

### 2.2. Materials

All participants underwent a neuropediatric and neuropsychological assessment, including the German Wechsler intelligence scales for children (WISC-IV) [29] and the Test of Variables of Attention (T.O.V.A.®) [28]. Additionally, functional aspects of attention were measured with a behavioral rating scale for parents (Conners 3®) [26]. Everyday aspects of executive functioning were measured with the questionnaire BRIEF® [27] (parent version).

The socio-economic status (SES) was measured with the Winkler-Index [30], taking into account the parents' educational achievement, their professional position and family income.

### 2.3. Data Analyses

Data were analyzed using the 25th version of the IBM Statistical Package for Social Science (SPSS). Group (NF1^ADHD^; NF1^control^; ADHD^control^) served as between subject factor and full-scale IQ, sex, and age as covariates, where appropriate. Due to high intercorrelation, full-scale IQ was excluded as covariate from the analysis on the WISC-IV subscales. The level of significance was set at *p* < 0.05 for all tests and adjusted by Bonferroni corrections for multiple comparisons. There were no relevant missing data (missing data: 8 participants for the SES, 4 participants for the Conners 3®), and the affected cases were excluded from the analyses.

To explore hypothesis one, data of the full-scale IQ and of all subscales of the WISC-IV (verbal comprehension, perceptual reasoning, working memory, and processing speed) were analyzed for group differences with two-tailed univariate and multivariate analyses of covariance ((M) ANCOVAs). Additionally, the correlation between full-scale IQ and attention performance index (API) of the T.O.V.A. was tested across both NF1 groups with a second-order partial correlation analysis (Pearson's correlation coefficient).

To test our second hypothesis, all parameters of the T.O.V.A. (Response Time Variability, Response Time, Commission Errors, and Omission Errors) and the subscales Inattention and Hyperactivity/Impulsivity of the Conners 3® were tested for group differences with two-tailed multivariate analyses of covariance (MANCOVAs). The global executive composite (GEC) score (=total score), the Behavioral Regulation Index, and the Metacognition Index of the BRIEF® were tested for group differences with a two-tailed univariate and a two-tailed multivariate analysis of covariance ((M)ANCOVAs). Additionally, frequencies of subnormal performances (scores <85) on the parameters of the T.O.V.A. were compared between the ADHD patient groups with nonparametric chi-square tests. NF1 patients without ADHD were included in the analyses as a control group.

## 3. Results

Table 1 summarizes the data of the explorative analyses for group characterization. There were no significant differences between the groups regarding age and SES, while sex distribution differed significantly between the groups. ADHD subtype did not differ significantly between the two ADHD groups. Patient groups differed significantly on the ADHD-index and the Global-index of the Conners 3®, with significantly higher scores for both ADHD groups than for the NF1^control^ group and significantly more profound ADHD symptoms in the ADHD^control^ group than in the NF1^ADHD^ group.

**Figure 1:**
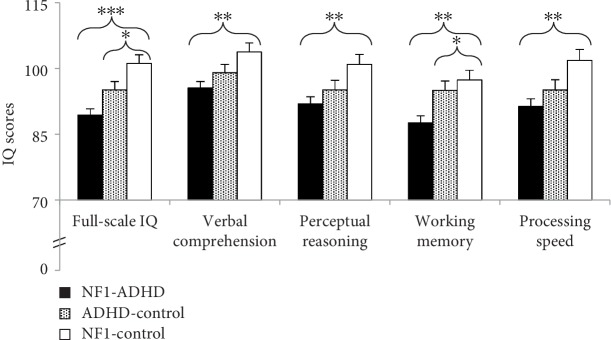
Intellectual profile of the intelligence test WISC-IV; standard scores (mean = 100; SD = 15).

Regarding the intellectual profile, there was a significant main effect for group on full-scale-IQ (*F*(2, 111) = 12.031, *MSE* = 103.06, *p* < .001), while the covariates sex and age did not have a significant effect on the dependent variable. Planned pairwise comparisons showed that the NF1^ADHD^ group scored significantly lower than the NF1^control^ group and the ADHD^control^ group (Figure 1).

The MANCOVA for the WISC-IV subscales revealed a significant effect of the covariate sex on processing speed (*F*(1, 111) = 8.064, *p* = .005), with girls performing better on processing speed than boys in both ADHD groups. After correcting for the effect of the covariate, significant main effects were observed for all four WISC-IV subscales (verbal comprehension: *F*(2, 111) = 5.498, *MSE* = 106.80, *p* = .005; perceptual reasoning: *F*(2, 111) = 5.094, *MSE* = 137.36, *p* = .008; working memory: *F*(2, 111) = 7.858, *MSE* = 131.03, *p* = .001; processing speed: *F*(2, 111) = 5.842, *MSE* = 163.78, *p* = .004), with the NF1^ADHD^ group performing significantly worse than the NF1^control^ group on all subscales and also performing worse than the ADHD^control^ group on the subscale working memory (Table 2). Between the NF1^control^ group and the ADHD^control^ group, there were no significant differences.

**Figure 2:**
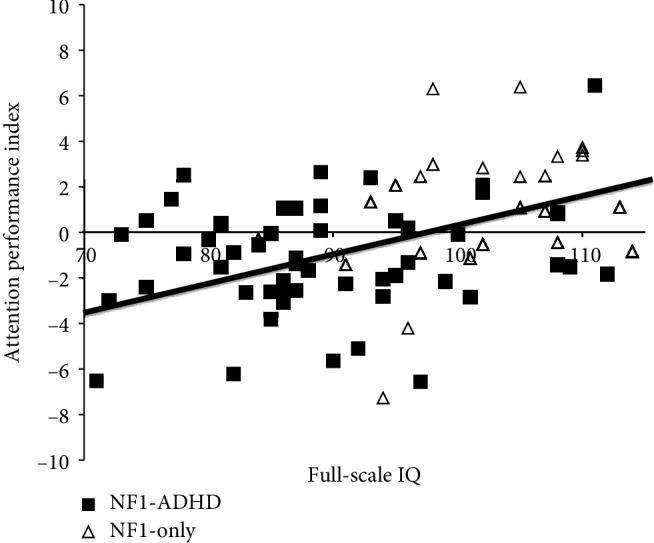
Correlation between full-scale IQ and attention performance index for combined NF1 sample.

The bias corrected second-order partial correlation analysis revealed a significant positive relationship between the API and the full-scale IQ (*r* = .408, BCa CI [.255, .549], *p* < .001) for the combined NF1 sample (Figure 2).

**Figure 3:**
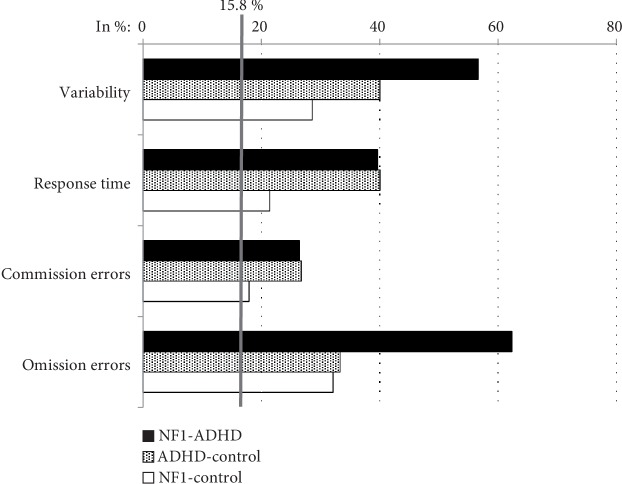
Frequencies of subnormal performance in percent.

The MANCOVA for the attention profile showed significant effects of the covariates full-scale IQ (*F*(4, 111) = 5.208,*p* = .001) and sex (*F*(4, 111) = 7.842, *p* < .001). After accounting for the effects of the covariates, there was no significant main effect of group on the parameters of the T.O.V.A. (Table 2). The comparison of frequencies of subnormal performances on the parameters of the T.O.V.A. showed significantly more reduced performances in the NF1^ADHD^ group on Omission Errors compared to the ADHD^control^group (*Pearsonχ* ^2^ (1, *N* = 83) = 6.422, *p* = 013). Also, the NF1^ADHD^ group showed significantly more reduced performances on response time variability (*Pearsonχ* ^2^ (1, *N* = 81) = 5.781, *p* = .020) and omission errors (*Pearsonχ* ^2^ (1, *N* = 81) = 6.658, *p* = .011) than the NF1^control^group. Between the ADHD^control^ group and the NF1^control^group, there were no significant differences on any parameter of the T.O.V.A. regarding the frequencies of subnormal performances (Figure 3).

For the Conners 3®, data of 4 patients were missing. In the analysis of the Conners 3®, there was a significant effect of the covariate sex on inattention (*F*(1, 107) = 8.607, *p* = .004), with girls being rated as more inattentive than boys in both ADHD groups. Full-scale IQ and age were not related to group differences on the Conners 3® subscales. After correcting for the effect of sex, significant main effects for group were found on the subscales inattention (*F*(2, 107) = 37.877, *MSE* = 34.87, *p* < .001) and Hyperactivity/Impulsivity (*F*(2, 107) = 13.146, *MSE* = 79.06, *p* < .001) of the Conners 3®. Planned pairwise comparisons showed that both ADHD groups were rated significantly worse than the NF1^control^ group on Inattention and Hyperactivity/Impulsivity, but did not differ significantly from each other (Table 2).

For the BRIEF®, data of 33 patients (24 NF1^ADHD^, 9 NF1^control^, and 0 ADHD^control^) were missing, because the questionnaire BRIEF® was not applied to patients that were assessed in the University Medical Center Hamburg-Eppendorf. The analyses of the BRIEF® scales showed a significant effect of group for the GEC score (*F*(2, 78) = 15.382, *MSE* = 93.30, *p* < .001) and for the behavioral regulation index (*F*(2, 78) = 6.117, *MSE* = 133.85, *p* = .004), as well as the metacognition index (*F*(2, 78) = 17.843, *MSE* = 97.06, *p* < .001). The covariates full-scale IQ, sex, and age had no influence on the dependent variables. Planned pairwise comparisons revealed that the NF1^control^ group was rated as significantly better than both other groups on the GEC score and the metacognition index. Additionally, the NF1^control^ group was rated as significantly better on the behavioral regulation index than the ADHD^control^ group (Table 2). Between the NF1^ADHD^ group and the ADHD^control^ group, there were no significant differences.

## 4. Discussion

Our study on the intellectual and attention profiles of NF1^ADHD^, NF1^control^, and ADHD^control^ yielded two main findings: Firstly, as predicted by our first hypothesis and complementing previous publications, the NF1 group in our sample could be divided in two distinct subgroups: A comorbid diagnosis of ADHD does seem to make a marked difference in the profile of intellectual and attention performance in patients with NF1. More specifically, an index of attention performance was closely associated with intellectual performance in NF1. Secondly, we could identify some qualitative differences between the attention performance of ADHD-patients with and without NF1, and also between NF1-patients with and without ADHD.

### 4.1. Intellectual Profile

Consistent with previous research [13, 17], we found that patients with NF1^ADHD^ showed the lowest IQ scores of all three groups and differed significantly from patients with NF1^control^in all measured areas of intellectual ability (Figure 1). Even though mean scores of all scales of the intelligence test lay within normal limits for the NF1^ADHD^ group, there seem to exist intellectual challenges in this group, which may not represent a serious disability, but could still crucially influence long-term adaptive functioning and participation. Furthermore, in NF1, reduced intellectual functioning correlated with reduced attention functions as measured with the T.O.V.A. attention performance index (API). From this finding we conclude that even subclinical attention problems are negatively associated with intellectual performance (Figure 2). While this correlation does not have the potential to reflect the multidimensional interactions between specific attention functions with specific intellectual functions, it provides a hint that the intellectual difference between the two NF1-groups is not just the result of behavioral or motivational problems inherent to the ADHD-condition, but that there is a true neuropsychological correlate which needs to be investigated more thoroughly.

Regarding the profile of intellectual abilities, we found an uneven profile in patients with NF1, similarly to Potvin et al., [17]. The NF1^ADHD^ group showed a parallel pattern to the NF1^control^ group with better verbal than visual-spatial skills and processing speed, and with the most intense weakness in working memory. These findings differ slightly from the results of Potvin and colleagues. They found the most pronounced weakness in processing speed in patients with NF1-only (and with ADHD-only).

In the present study, working memory had the lowest scores of the subscales of the WISC-IV in all three patient groups, but the performance was far more decreased in the NF1^ADHD^ group. In the NF1^ADHD^ group, 47.2 percent showed impaired working memory skills (scores below the normal range) compared to 10.7 percent in the NF1^control^ group and 10.0 percent in the ADHD^control^ group. Our results suggest that problems in executive functioning and short-term memory are associated with NF1, but also that these problems are exacerbated by additional ADHD symptoms. Since our measures on other executive functions apart from working memory were retrieved from questionnaires, a differentiated profile of executive deficits could not be evaluated, and our assumptions must stay on a global level. However, a simple summation of negative effects of NF1 and ADHD symptoms on working memory skills in NF1^ADHD^ is unlikely considering the low numbers of patients with NF1-only and ADHD-only showing reduced working memory skills in our study. Apparently it is the combination of NF1 and ADHD that leads to an additional cognitive burden.

One very interesting finding of our study is that patients with NF1^control^ scored, as a group, at the population mean of intellectual functioning, which somewhat contradicts the consistent findings of earlier research of generally reduced intellectual functioning in NF1 [3]. However, most prior studies reporting a generalized downward shift of IQ in NF1 treated the population as one homogeneous group. More recent studies, however, considered a possible negative influence of attention deficit on intellectual functioning and therefore differentiated between patients with and without ADHD within the NF1 population. These studies indicate that ADHD is a potential risk factor for intellectual dysfunction. Two retrospective studies analyzing clinical patient data provided first evidence for an additional cognitive burden of patients with NF1^ADHD^ [13, 17]. Our study now confirmed and extended these results prospectively in a more representative sample. One possible interpretation of this finding is a direct influence of attention problems on the acquisition of skills and knowledge, or, even more simply, on the performance in intelligence tests. However, given the multi-faceted neurocognitive profile of NF1, and the complexity of the attention domain, this assumption is likely insufficient. With attention deficit being only one in a spectrum of NF1-related neurodevelopmental problems, a common cause in the neurobiological basis of NF1 is highly probable, leading to a range of cognitive deficits down the stream of aberrant neural pathways and altered neurotransmitter systems. The combination of these two aspects, supplemented by adaptive developmental processes, might account for the apparently categorical distribution of cognitive profiles between NF1 with or without ADHD symptoms.

### 4.2. Attention Profile

In the neurotypical population, ADHD is a heterogeneous condition with a variety of profiles in attention and executive functions [31]. Assuming that ADHD symptoms are the endpoint of a more uniform neurobiological pathway in NF1, we expected both NF1 groups to exhibit a more concise profile of attention and executive functions. At first sight, however, data of the T.O.V.A. and of the parent rating scales (Conners 3® and BRIEF®) provide a comparable picture in the two ADHD groups with moderate inattention, mild hyperactivity/impulsivity, and mild executive dysfunction. The attention profile of the NF1^control^ group reveals mild inattention (i.e., a subnormal mean score on omission errors in the T.O.V.A.), but no deficits in hyperactivity/impulsivity or executive functions. No significant differences between mean scores of specific attention domains were found. However, a closer look into the samples reveals a more detailed picture.

Both ADHD groups presented slightly subnormal mean scores on response time variability (reflecting distractibility) and omission errors (reflecting inattention), but additionally, the ADHD^control^ group showed a reduced performance on response time. These results are in line with the findings of a recent study, where neurotypical patients with ADHD showed inferior response times to patients with NF1^ADHD^ in a sustained attention task [23].

The frequencies of subnormal performances show an important and significant difference between patients with ADHD with and without NF1 in the T.O.V.A. Nearly twice as many patients with NF1^ADHD^ (62.3%) than patients with ADHD^control^ (33.3%) showed subnormal performances on Omission Errors (reflecting inattention). Response Time Variability was also more often reduced in the NF1^ADHD^ group than in the ADHD^control^group, even though there were no significant differences.

Overall, the significantly higher number of patients with impaired performance on Omission Errors in the NF1^ADHD^ group is also reflected in the mean scores and these results together lead to the strong assumption that the attention profiles of patients with NF1^ADHD^differ from those of patients with ADHD^control^. Patients with NF1^ADHD^ seem to be especially affected regarding inattention, as is the case with patients with NF1 without ADHD. Our results confirm previous research showing that symptoms of inattention are more prevalent in NF1 [14] and they support the idea that certain deficits are rather associated with NF1 than merely the result of an comorbid ADHD symptomatology [32, 33].

Even if there is still a marked difference between NF1 patients with and without an additional ADHD diagnosis, the number of below average performances in the NF1^control^ group is high, especially in the areas of distractibility and inattention. Furthermore, there were no statistical differences between patients with NF1-only and patients with ADHD-only in the frequencies of subnormal performances. Altogether, this leads to the conclusion that subclinical attention problems are prevalent among patients with NF1 without an ADHD diagnosis and might be an inherent cognitive feature of the NF1 condition. Affected inattention/sustained attention in patients with NF1 was found before [14], but affected performances in other areas of attention functions are an unexpected and novel finding that requires further investigation.

The heterogeneity of the incidence of subclinical attention problems or ADHD in the NF1 population might be explained by different phenotype expressions. Even if NF1 is a monogenetic disorder, this does not mean that it is a homogeneous medical condition. Evidence rather suggests that factors like sex, age, specific cell type, genomic modifiers, and micro-environmental influences determine the cognitive and behavioral phenotype triggered by the NF1 condition. Different levels of *Ras (Rat sarcoma)* and dopamine activity in specific combinations are proposed to contribute to diverse cognitive profiles in NF1: High levels of *Ras* activity plus slightly reduced levels of dopamine might lead to severe spatial learning and memory deficits, while high levels of *Ras* activity plus heavily reduced levels of dopamine might result in severe attention problems and mild learning deficits [10]. Additionally, to elucidate the role of *Ras*, dopamine or other neurotransmitters on learning, memory, and attention deficits in NF1, further research is needed.

### 4.3. Limitations

Some limitations have to be considered in the interpretation of our results. We acknowledge that we partly recruited patients, who had an indication for clinical neuropsychological diagnostics because of developmental, behavioral, or academic problems. Therefore, the intellectual and attention performance of these patients might be worse than that of other patients with NF1 or ADHD. Additionally, we excluded NF1 patients with intracranial complications, which might limit the representativeness of our NF1 patient group. However, since intracranial complications in NF1 (e.g., intracranial tumors with 1-2% or hydrocephalus with 2% [34]) are rare, the results of our study should still be valuable for the majority of patients with NF1.

Furthermore, we used only one continuous performance test and one attention questionnaire to assess the attention profile of our patients. Although the T.O.V.A. test is very good in predicting ADHD in individuals, it might not measure specific attention functions differentiated enough to distinguish between ADHD in NF1 and ADHD-only. For further studies on this topic the use of more elaborate test batteries is recommended, to address different domains of attention and also executive functions and to gain a more detailed picture of the profiles of ADHD in NF1 and ADHD-only.

Regarding executive dysfunctions, there is a limitation in that we only used external ratings of everyday functioning and no test measures to assess them. As a consequence, we have a high rate of missing data for this area. For a comprehensive evaluation of executive functions further studies should include both types of measurement, since it is well known that data of external ratings and test measures have the tendency to differ from each other [35] and are suspected to tap different executive functions [36].

Another limitation is that we investigated three patient groups of very different group sizes, which might limit the statistical power of our analyses and might affect type II error rates. Additionally, the ADHD^control^ group was rather small considering the prevalence of ADHD. Therefore, it was not possible to differentiate between ADHD subtypes in our patient groups. Even though the distribution of subtypes did not differ significantly between the NF1^ADHD^ group and the ADHD^control^ group, slight differences in the frequency of the subtypes could have had an effect on the results. Moreover, we cannot contribute to the open questions of diverse distributions of ADHD symptoms and ADHD subtypes in NF1 and ADHD^control^. Also, the sex distribution was uneven in all three patient groups and was nearly reversed in the ADHD^control^ group compared to the NF1^control^ group. Even though we used sex as a covariate in our analyses, the qualitative differences between boys and girls regarding ADHD symptomatology and eventually also cognitive dysfunction in NF1 could still have influenced our results.

## 5. Conclusion

First of all, our findings support the hypothesis that NF1 per se might not lead to globally impaired intellectual functioning, but that attention deficits are a specific additional risk factor for suboptimal intellectual performance in NF1, like ADHD is in the general population [15]. Second, our results show that the frequency of deficits in specific attention domains of NF1^ADHD^ differs from that of ADHD-only, which goes along with a different distribution of ADHD subtypes in the two groups. Both findings indicate that ADHD in NF1 is not only a simple comorbidity and that the condition NF1^ADHD^ comes with an additional cognitive burden. Furthermore, there seem to exist (subclinical) attention deficits in NF1, which are rather associated with the NF1 condition than with a comorbid ADHD diagnosis. Assuming that there are—at least–two distinct profiles of NF1 with one group being more affected than the other, this would encourage further investigations on neurobiological causes of cognitive dysfunction in NF1 and entail implications for potential treatment options [11, 37].

## Figures and Tables

**Table 1:** Sample characterization of patients with NF1^ADHD^, NF1^control^, and ADHD^control^.

	Corrected means (standard error)								
	NF1^ADHD^ (A)	NF1^control^ (B)	ADHD^control^(C)	*F* (all groups)*^a^*	*MSE*	*p* values (all groups)	Post-hoc comparisons^a^		
Number	53	28	30	—	—	—	—		
Sex (female/male)	20/33	18/10	8/22	—	—	0.010^c^	—		
Age	8.87 (0.20)	8.36 (0.28)	8.96 (0.27)	1.454	2.175	0.238^d^	—		
SES (Winkler-index)^b^	12.02 (0.64)	13.39 (0.86)	11.13 (0.80)	1.88	18.99	0.159^d^	—		
Familial type of NF1 (familial/spontaneous)	25/28	9/19	—	—	—	0.240^c^	—		
Asymptomatic optic nerve glioma (N)	10	3	—	—	—	0.358^c^	—		
Subtype (ADHD-C/ADHD-I)^f^	33/20	—	16/14	—	—	0.490^c^	—		
Methylphenidate prior to T1 (N)	7	—	2	—	—	0.292^c^	—		
Conners 3® ADHD-index (*T*-scores)	62.48 (0.86)	52.93 (1.27)	66.05 (1.07)	31.29	33.58	<0.001^∗∗∗^^e^	A<B^∗∗∗^; C<B^∗∗∗^; C<A^∗^		
Conners 3® global-index (*T*-scores)	60.22 (1.16)	49.61 (1.72)	65.03 (1.46)	23.17	61.78	<0.001^∗∗∗^^e^	A<B^∗∗∗^; C<B^∗∗∗^; C<A^∗^		
Allowed comorbidities	Number of patients					*p* values (all groups)	Post-hoc comparisons^a^		
Previous language disorder	14	4	7	—	—	.415^c^	—		
Unspecific learning disabilities	9	1	1	—	—	.108^ c^	—		
Dyslexia	8	1	6	—	—	.188^ c^	—		
Dyscalculia	5	0	3	—	—	.519^ c^	—		
Depression	0	0	1	—	—	.538^ c^	—		
Anxiety disorders	1	1	1	—	—	.457^ c^	—		
Oppositional defiant disorder	4	0	3	—	—	.325^ c^	—		
Conduct disorder	2	0	2	—	—	.325^ c^	—		

*Note:* ^a^< = “worse than”, even if the score is higher in numbers; ∗^*p*^ < .05; ∗∗^*p*^ < .01; ∗∗∗^*p*^ < .001; ^b^The socio-economic status was measured with the Winkler-Index (3–8 = low, 9–14 = middle, 15–21 = high).

^c^Data was analyzed with *χ* ^2^-tests; ^d^Data was analyzed with analyses of variance (ANOVA); ^e^Data was analyzed with multivariate analyses of covariance (MANCOVA). ^f^ADHD-C = combined type of ADHD, ADHD-I = inattentive type of ADHD.

**Table 2:** Attention profile and intellectual profile of patients with NF1^ADHD^, NF1^control^, and ADHD^control^.

	Corrected means (standard error)							
	NF1^ADHD^ (A)	NF1^control^ (B)	ADHD^control^(C)	*F* (all groups)*^a^*	*MSE*	*p* (all groups)	*η^2^*	Post-hoc comparisons^b^
WISC-IV (standard score)				*F*(2, 111)				
Full-scale IQ	89.39 (1.40)	101.14 (1.98)	95.12 (1.88)	12.031	103.06	<0.001^∗∗∗^	0.185	A<B^∗∗∗^; A<C^∗^
WISC-IV subscales (standard scores)				*F*(2, 111)				
Verbal comprehension	95.58 (1.42)	103.77 (2.02)	99.03 (1.91)	5.498	106.80	0.005^∗∗^	0.094	A<B^∗∗^
Perceptual reasoning	91.93 (1.61)	100.93 (2.29)	95.12 (2.17)	5.094	137.36	0.008^∗∗^	0.088	A<B^∗∗^
Working memory	87.62 (1.58)	97.37 (2.24)	95.03 (2.12)	7.858	131.03	0.001^∗∗^	0.129	A<B^∗∗^, A<C^∗^
Processing speed	91.33 (1.76)	101.85 (2.50)	95.09 (2.37)	5.842	163.78	0.004^∗∗^	0.099	A<B^∗∗^
T.O.V.A. (standard scores)				*F*(2, 111)				
Variability	84.57 (2.54)	92.34 (3.65)	81.17 (3.62)	2.610	309.25	0.078	0.047	—
Response time	91.44 (2.56)	93.92 (3.68)	84.47 (3.29)	2.166	315.23	0.120	0.040	—
Commission errors	95.43 (2.60)	98.92 (3.74)	95.61 (3.35)	0.296	325.44	0.745	0.006	—
Omission errors	72.26 (3.28)	83.06 (4.71)	81.57 (4.21)	2.252	516.10	0.110	0.041	—
Conners 3® (*T*-scores)^c^				*F*(2, 107)				
Inattention	65.23 (0.87)	54.02 (1.29)	68.62 (1.09)	37.877	34.87	<0.001^∗∗∗^	0.429	A<B^∗∗∗^; C<B^∗∗∗^
Hyperactivity/impulsivity	59.56 (1.32)	48.80 (1.94)	61.41 (1.65)	13.146	79.06	<0.001^∗∗∗^	0.207	A<B^∗∗∗^; C<B^∗∗∗^
BRIEF® (*T*-scores)^d^				*F*(2, 78)				
GEC score	61.83 (1.92)	47.80 (2.48)	64.86 (1.79)	15.382	93.30	<0.001^∗∗∗^	0.999	A<B^∗∗∗^; C<B^∗∗∗^
Behavioral regulation index	57.08 (2.30)	47.87 (2.97)	60.87 (2.16)	6.117	133.85	0.004^∗∗^	0.876	C<B^∗∗^
Metacognition index	64.28 (1.96)	48.22 (2.53)	66.82 (1.84)	17.843	97.06	<0.001^∗∗∗^	1.000	A<B^∗∗∗^; C<B^∗∗∗^

*Note:* ^a^Full-scale IQ was analysed with an univariate analysis of covariance (ANCOVA), all other data was analyzed with multivariate analyses of covariance

(MANCOVAs); ^b^ **<** = “worse than”, even if the score is higher in numbers; ∗^*p*^ < .05; ∗∗^*p*^ < .01; *p* ^∗∗∗^ < .001; ^c^ For the Conners 3®, data of to 4 patients was missing;

^d^For the BRIEF®, data of 33 patients were missing (included were 29 NF1^ADHD^, 19 NF1^control^, and 30 ADHD^control^ patients).

## Data Availability

The statistical data used to support the findings of this study are included within the article. Additional statistical data or source files used to support the findings of this study are available from the corresponding author upon request.
